# Visual-Cerebellar Pathways and Their Roles in the Control of Avian Flight

**DOI:** 10.3389/fnins.2018.00223

**Published:** 2018-04-09

**Authors:** Douglas R. Wylie, Cristián Gutiérrez-Ibáñez, Andrea H. Gaede, Douglas L. Altshuler, Andrew N. Iwaniuk

**Affiliations:** ^1^Neuroscience and Mental Health Institute, University of Alberta, Edmonton, AB, Canada; ^2^Department of Zoology, University of British Columbia, Vancouver, BC, Canada; ^3^Department of Neuroscience, Canadian Centre for Behavioural Neuroscience, University of Lethbridge, Lethbridge, AB, Canada

**Keywords:** cerebellum, optic flow processing, lentiformis mesencephali, pontine nuclei, motion parallax, flight control

## Abstract

In this paper, we review the connections and physiology of visual pathways to the cerebellum in birds and consider their role in flight. We emphasize that there are two visual pathways to the cerebellum. One is to the vestibulocerebellum (folia IXcd and X) that originates from two retinal-recipient nuclei that process optic flow: the nucleus of the basal optic root (nBOR) and the pretectal nucleus lentiformis mesencephali (LM). The second is to the oculomotor cerebellum (folia VI-VIII), which receives optic flow information, mainly from LM, but also local visual motion information from the optic tectum, and other visual information from the ventral lateral geniculate nucleus (Glv). The tectum, LM and Glv are all intimately connected with the pontine nuclei, which also project to the oculomotor cerebellum. We believe this rich integration of visual information in the cerebellum is important for analyzing motion parallax that occurs during flight. Finally, we extend upon a suggestion by Ibbotson (2017) that the hypertrophy that is observed in LM in hummingbirds might be due to an increase in the processing demands associated with the pathway to the oculomotor cerebellum as they fly through a cluttered environment while feeding.

Gibson (1954) emphasized that self-motion results in optic flow across the entire retina and that such visual information can be used to control posture and locomotion. One of the classic illustrations from Gibson (1979) to exemplify this is reproduced in Figure 1, which shows the optic flowfield resulting from a bird in flight. Despite widespread evidence supporting the importance of optic flow in modulating posture and locomotion across vertebrate species (e.g., Lee, 1980; Warren et al., 2001), it is only recently that a few studies (Bhagavatula et al., 2011; Goller and Altshuler, 2014; Schiffner and Srinivasan, 2015; Dakin et al., 2016; Ros and Biewener, 2016) have explicitly demonstrated that manipulation of the optic flow field can affect flight behavior of birds (Lee and Reddish, 1981; but see, Davies and Green, 1990; Lee et al., 1991, 1993; Eckmeier et al., 2008). Optic flow is analyzed by specialized visual pathways in the avian brain, which originate from two retinal-recipient nuclei: the nucleus of the basal optic root (nBOR) of the Accessory Optic System, and the pretectal nucleus lentiformis mesencephali (LM). Neurons in LM and nBOR have very large receptive fields and exhibit direction-selectivity in response to optic flow stimuli (Burns and Wallman, 1981; Morgan and Frost, 1981; Winterson and Brauth, 1985; Wylie and Crowder, 2000). Such responses are unique to LM and nBOR cells, as other motion-sensitive cells in the visual system respond best to small object-like stimuli, and have large-inhibitory surrounds such that they do not respond to optic flow (Frost et al., 1990). Although LM and nBOR have been implicated in the generation of the optokinetic response for retinal image stabilization (Fite et al., 1979; Gioanni et al., 1983a,b), their involvement in the visual control of flight has yet to be demonstrated. However, Iwaniuk and Wylie (2007) noted that the LM was 2 to 5X larger in hummingbirds compared to other birds (Figures 2A,B). LM was also enlarged in species that occasionally hover, such as the eastern spinebill (*Acanthorhynchus tenuirostris*), a nectarivorous Australian songbird. As hovering represents a specialized case of stabilization, Iwaniuk and Wylie (2007) postulated that the hypertrophy of the LM was to facilitate hovering. Subsequently, Gaede et al. (2017) recorded from the LM in hummingbirds and noted their response properties to largefield stimuli were quite different from other birds. nBOR and LM have extensive direct and indirect connections with the cerebellum (Clarke, 1977; Brecha et al., 1980; Gamlin and Cohen, 1988a; Wylie et al., 1997; Pakan and Wylie, 2006), which is well established to be involved in motor control (Ito, 1984). Pakan and Wylie (2006) noted that nBOR and LM give rise to two optic flow pathways to the cerebellum; to the posterior part, folia IXcd and X, which collectively are know as the vestibulocerebellum (VbC), and to the oculomotor cerebellum, which is comprised of folia VI-VIII (see below). In this paper, we will expand upon the proposed role of the LM and visual cerebellar pathways in avian flight by exploring several questions: What other visual nuclei should show hypertrophy in concert with the LM? Which part of the LM is hypertrophied in hummingbirds? And finally, is the expansion of the LM in hummingbirds driven by the visual demands of behaviors other than hovering, as preciously suggested?

**Figure 1:**
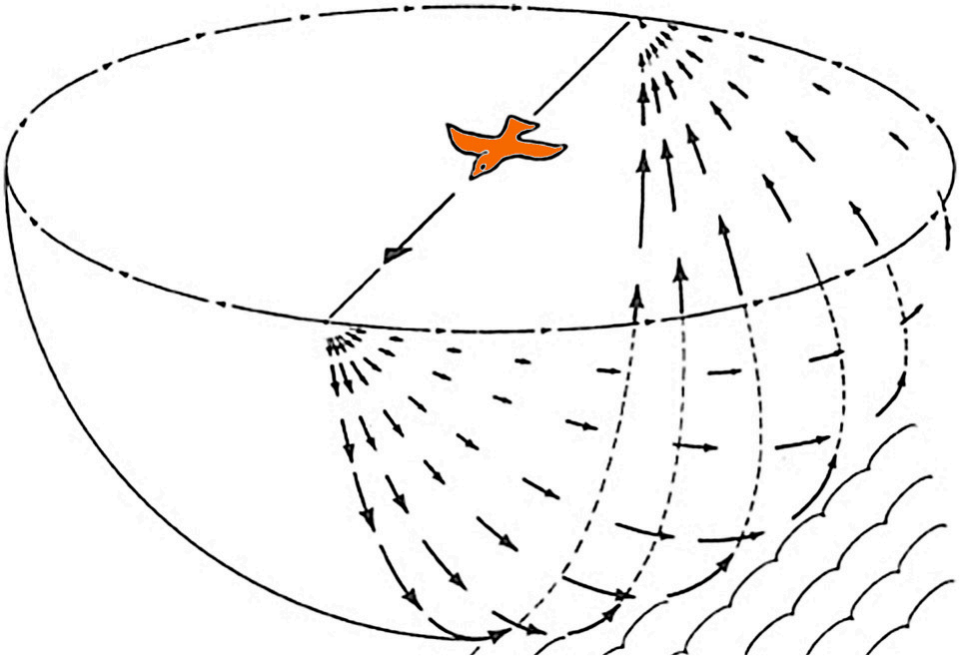
The optic flowfield during forward flight. Adapted from Gibson (1979).

**Figure 2:**
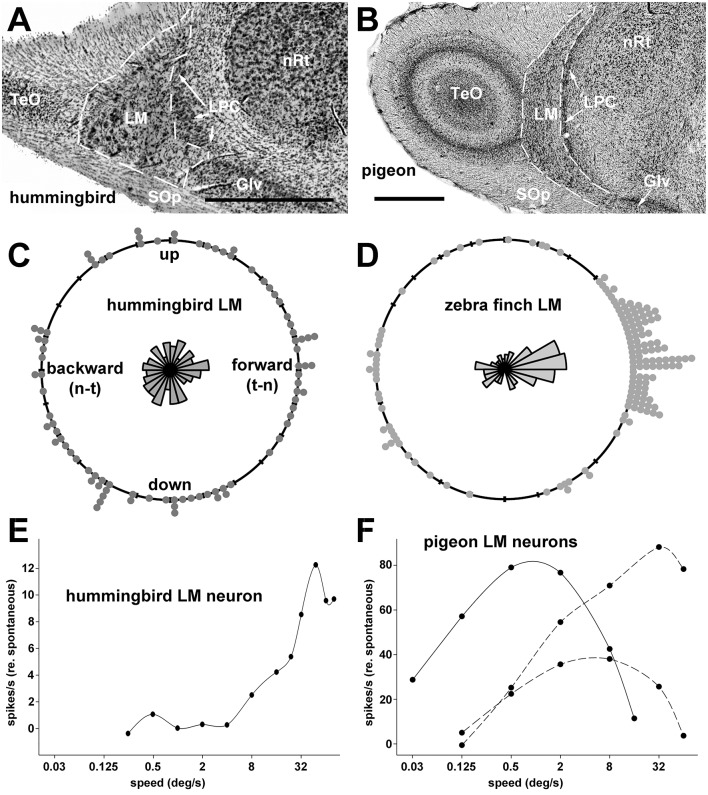
Coronal sections through the pretectal nucleus lentiformis mesencephali (LM) in a hummingbird (*Doryfera ludoviciae*) (**A**, from Iwaniuk and Wylie, 2007, with permission) and a pigeon (*Columba livia*) **(B)**. **(C,D)** Respectively show the direction preferences of LM neurons in Anna's hummingbird (*Calypte anna*) and zebra finch (*Taeniopygia guttata*) (from Gaede et al., 2017, with permission). **(E)** Shows the speed tuning curve for a LM neuron in a Anna's hummingbird (from the data set of Gaede et al., 2017). The visual stimuli were largefield random dot patterns moving in the preferred direction. **(F)** Shows the speed tuning for 3 LM neurons in pigeon: (from the data set of Crowder et al., 2003). One is a “slow” neuron that preferred forward (nasal to temporal) motion (solid line), whereas the other two are “fast” neurons that preferred backward (lower dashed line) and downward (upper dashed line) motion. The visual stimulus were largefield sine wave gratings of an effective spatial frequency (1 cycle per degree [cpd] for the slow neuron, 0.25cpd for the fast neurons) drifting in the preferred directions. Other abbreviations: Glv, ventral lateral geniculate nucleus; LPC, nucleus laminaris precommissuralis; nRt, nucleus rotundas; TeO, optic tectum; SOp, stratum opticum; t-n, temporal to nasal; n-t, nasal to temporal. Scale bars; 0.5 mm in **(A)** 1 mm in **(B)**.

## Response properties of neurons in nBOR and LM

Neurons in nBOR and LM are ideally suited for the analysis of optic flow because they have large receptive fields averaging about 60° in diameter with some encompassing the entire monocular visual field. These neurons are directionally selective in response to large stimuli, such as random dot patterns, checkerboards, and gratings. The response properties of nBOR neurons have been investigated in chickens (*Gallus gallus*, Burns and Wallman, 1981) and pigeons (Morgan and Frost, 1981; *Columba livia*, Gioanni et al., 1984; Wylie and Frost, 1990), and the responses are essentially identical. With respect to direction preference, neurons that prefer upward, downward and backward (i.e., nasal-to-temporal) motion are about equally abundant in nBOR, but fewer (5–10%) prefer forward (i.e., temporal-to-nasal) motion (Frost et al., 1990). With respect to stimulus speed, neurons are broadly tuned, but the majority (~75%) prefer slow velocities (< 5°/s) (Burns and Wallman, 1981; Crowder et al., 2003). Data from chickens, pigeons and zebra finches (*Taeniopygia guttata*) suggest that the LM is complementary to the nBOR regarding direction preference. In LM, there is a clear directional bias to forward motion: about half the neurons prefer motion in this direction and neurons that prefer upward, downward and backward motion are equally represented (McKenna and Wallman, 1985b; Winterson and Brauth, 1985; Wylie and Frost, 1996; Wylie and Crowder, 2000; Gaede et al., 2017) (Figure 2D). The bias toward neurons that prefer forward motion has also been found in the homologs of the LM in amphibians, reptiles, and mammals (Collewijn, 1975; Katte and Hoffmann, 1980; Fan et al., 1995). With respect to speed, as in nBOR, LM neurons are broadly tuned, but most (about 65%) prefer faster stimuli (>5°/s and up to 250°/s). Almost all (>95%) of the LM neurons that prefer slower speeds prefer forward motion. Stated another way, there are two groups of LM neurons: (i) slow neurons that prefer forward motion; and (ii) fast neurons that prefer upward downward and backward motion (Wylie and Crowder, 2000) (see Figure 2F). There is also evidence suggesting that there is a separation of directional response types within LM. Winterson and Brauth (1985) noted that most cells in the LM parvocellularis, now known as the lateral LM (LMl; Gamlin and Cohen, 1988b) preferred forward motion, whereas cells that prefer upward, downward, backward and forward motion are found in LM magnocellularis, now known as the medial LM (LMm, Gamlin and Cohen, 1988b). This is important because the LMm and LMl project to different parts of the cerebellum (see below, Pakan and Wylie, 2006).

## The LM in hummingbirds

As mentioned above, LM is hypertrophied in hummingbirds (Iwaniuk and Wylie, 2007). In a more recent study, Gaede et al. (2017) recorded from the LM of Anna's hummingbird (*Calypte anna*) and noted that the neuronal responses to largefield visual stimuli differed in hummingbirds compared with other bird species. First, a directional bias to forward motion was not found, rather all directions were represented equally (Figure 2C). Second, LM neurons in hummingbirds were tightly tuned to stimulus velocity, and mainly to speeds higher than 48°/s (Figure 2E). In this regard, hummingbirds could be regarded as visual “velocity specialist,” forgoing coarse coding common in perceptual systems for a specificity code seen in other sensory specialists, such as neurons tuned for auditory space in owls (Konishi, 2003; Lesica et al., 2010). Insofar as Winterson and Brauth (1985) noted that LMl contained neurons that prefer forward motion, whereas neurons representing all directions of motion, (which prefer faster speeds), were found in LMm, we speculate that the hypertrophy of the LM in hummingbirds may be largely due to an expansion of the LMm.

## The cerebellum and flight

**Figure 3:**
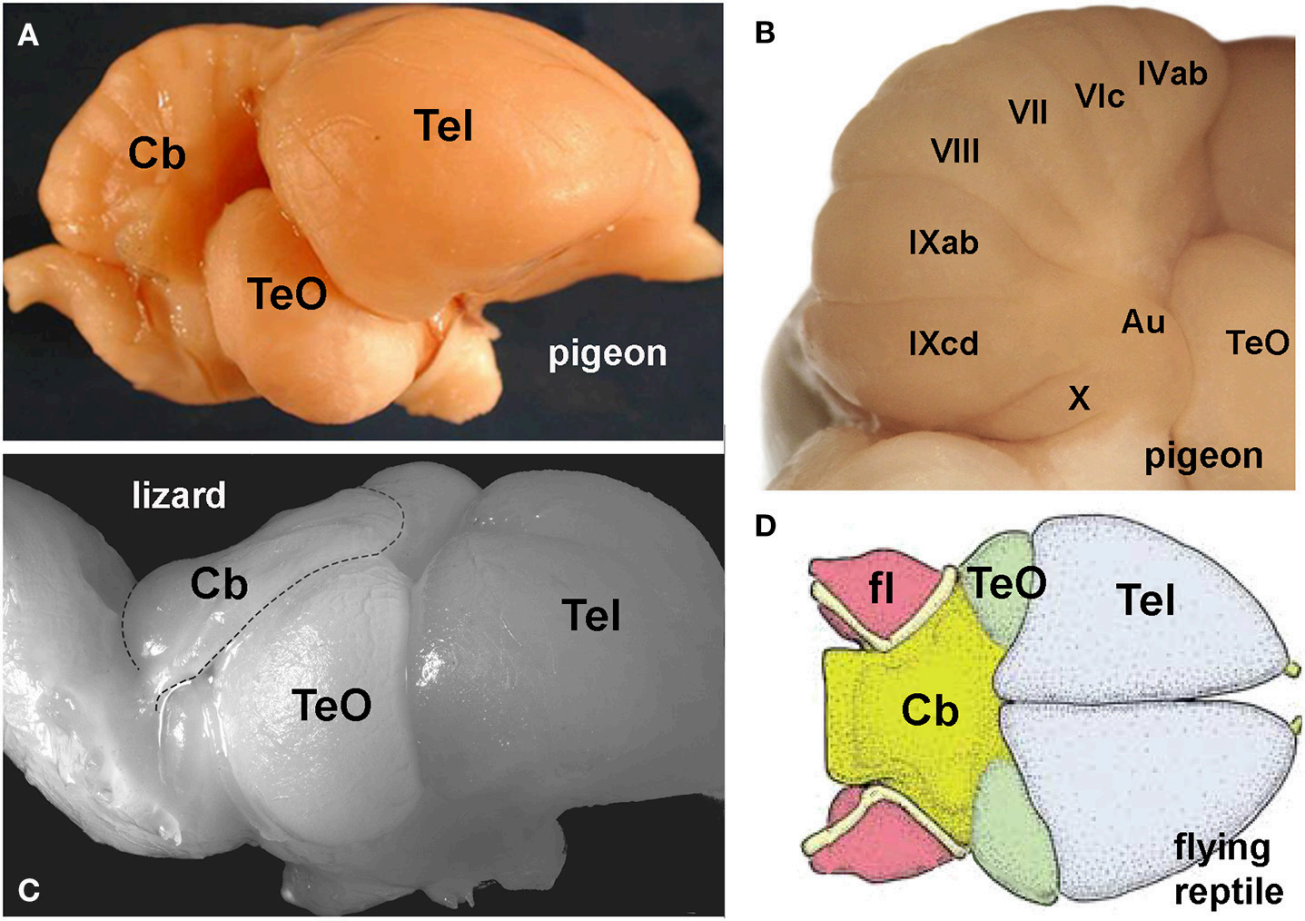
**(A)** Shows a lateral view of the pigeon brain, to emphasize the relatively large size of the cerebellum (Cb). **(B)** Shows the numbered folia of the posterior lobe. The vestibulocerebellum (VbC) includes folia IXcd and X, and the laterally protruding auricle (Au). **(C)** Shows a lateral view of the brain of a dragon lizard (*Ctenophorus nuchalis*) emphasizing the small underdeveloped Cb) (from Wylie et al., 2016, with permission). **(D)** Shows a dorsal view of the brain (endocast) of a flying reptile (*Rhamphorhynchus muensteri*) showing the large cerebellum (yellow) and the cerebellar flocculus (fl) (from Witmer et al., 2003, with permission). Other abbreviations: TeO, optic tectum; Tel, telencephalon.

**Figure 4:**
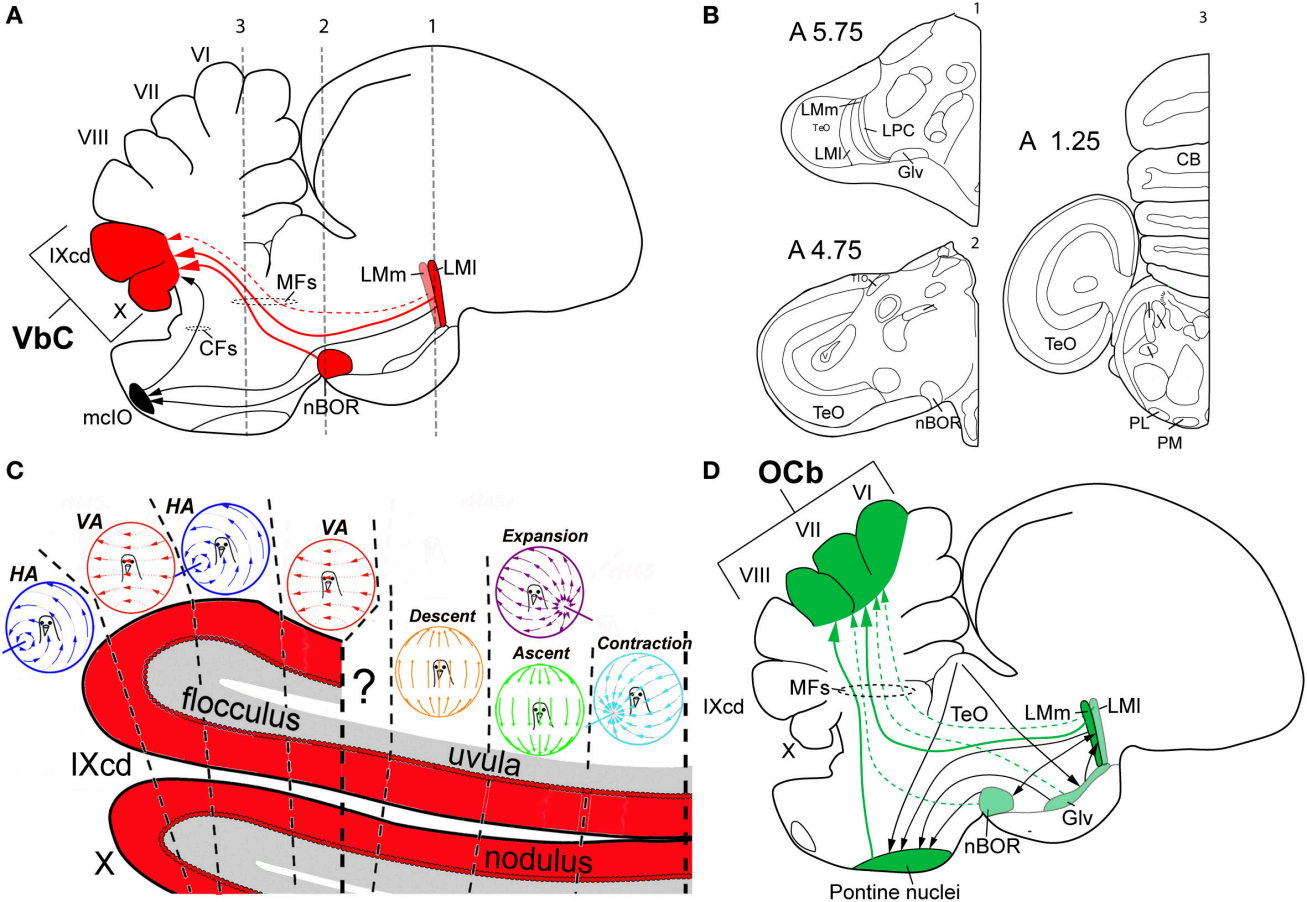
Visual cerebellar pathways in birds. **(A)** Shows in red the optic flow pathways from the nucleus of the basal optic root (nBOR) and pretectal nucleus lentiformis mesencephalic (LM) to the vestibulocerebellum (VbC; folia IXcd and X), black arrows show projections to the medial column of the inferior olive (mcIO). **(B)** Shows schematic drawings of coronal sections at three different anteroposterior levels (1–3 in **A**) of the pigeon's brain to show the relative position of the different nuclei involve in visual pathways to the cerebellum in birds. Coordinates in the pigeon brain atlas of Karten and Hodos (1967) are shown. Adapted from Karten and Hodos (1967). **(C)** Shows the sagittal optic flow “zones” in the VbC. **(D)** Shows in green and black the extensive pretecto-ponto-cerebellar connectivity to the oculomotor cerebellum (OCb, folia VI–VII). The larger arrows with solid lines represent heavier projections, whereas the smaller arrows with dotted lines represent weaker projections. See text for details. Other abbreviations: CFs, climbing fibers; Glv, ventral lateral geniculate nucleus; HA, horizontal axis (neurons); LMl, lateral LMm, medial LM; MFs, mossy fibers; PL, lateral pontine nucleus; PM, medial pontine nucleus; VA, vertical axis (neurons).

It is not unreasonable to assume that flight demands acute multisensory integration and sophisticated motor control. The cerebellum is large and well developed in birds (Figure 3A) and as the cerebellum is traditionally implicated in motor control (Ito, 1984) and is clearly a site of multisensory integration (Paulin, 1993; Bower, 1997) it is not surprising that it is thought to be important for flight (Kornhuber, 1974). Husband and Shimizu (2001) noted that the cerebellum is much larger in birds compared to non-avian reptiles (Figures 3A–C). Witmer et al. (2003) examined gross brain morphology from the endocasts of extinct flying reptiles and noted that there was a large cerebellum, in particular, the flocculus (fl) of the VbC (Figure 3D). However, Walsh et al. (2013) urge caution in this regard, because they found no correlation between the flying behavior and the size of the flocculus in extant birds, and large cerebella are also present in several bird-like non-flying dinosaurs. Nonetheless, Walsh et al. acknowledge that the evolution of the large cerebellum may have rendered birds flight ready. Finally, Feenders et al. (2008) used the expression of immediate-early genes as an indicator of neural activity during various behaviors. During flight, activity was evident, especially in folia VI and IXcd. As mentioned, optic flow pathways to the cerebellum in birds are precisely to these two areas, the oculomotor cerebellum (folia VI–VII) and the VbC (folia IXcd and X, see below, Figure 4). Thus, several lines of evidence suggest a key role for the cerebellum in avian flight.

## The optic flow pathway to the vestibulocerebellum

The first optic flow pathway to the cerebellum is to the VbC, folia IXcd and X (Figure 4A). The VbC is divided into the lateral flocculus (fl), which includes the lateral protrusion of the cerebellum known as the auricle (see Figure 3B), and the medial VbC comprised of the ventral uvula and nodulus. Optic flow information reaches the VbC directly, as mossy fibers (MFs) that originate from neurons in both nBOR and LM and terminate in folium IXcd, but not X (see Figures 4A,B for details and the relative position of each nuclei in the brain of the pigeon) (Clarke, 1977; Brecha et al., 1980; Gamlin and Cohen, 1988a). The majority (~75%) of the LM projection is from LMl (Pakan and Wylie, 2006). nBOR and LM also project indirectly to the VbC via the medial column of the inferior olive (mcIO), whose cells ascend to the VbC as climbing fibers (CFs) (Arends and Voogd, 1989; Lau et al., 1998; Wylie, 2001). This indirect projection from the LM arises from fusiform cells that lie in a thin strip along the border between LMm and LMl (Pakan et al., 2006). These two different pathways(CF and MF), also originate from cells with different speed preferences: the CF pathway originates from the slow cells in the nBOR and LM, whereas the MF pathway is fed by both slow and fast cells in nBOR and LM (Winship et al., 2005). This CF pathway to the VbC has been studied in detail in several mammalian species (e.g., Graf et al., 1988), and is critical for retinal image stabilization and the modification of the vestibular ocular reflex by visual-information (Ito, 1998). This is a highly conserved pathway, as the physiology and anatomy of this CF pathway to the flocculus is strikingly similar in birds and mammals (Voogd and Wylie, 2004).

The CFs give rise to the complex spike activity (CSA) of Purkinje cells in mammals, birds and likely other vertebrates (Eccles et al., 1966; Wylie and Frost, 1991). Depicted in Figure 4C, the VbC in birds is organized into several optic flow “zones” (Voogd and Bigaré, 1980), which lie in the sagittal plane and cut across IXcd and X. The CSA in the flocculus responds most strongly to rotational optic flow about either the vertical axis (VA neurons) or an horizontal axis oriented 45° to the midline (HA neurons) (Wylie and Frost, 1993). In pigeon, there are two VA zones interdigitated with two HA zones (Winship and Wylie, 2003). In the uvula/nodulus, the CSA responds most strongly to optic flow resulting from translation (Wylie et al., 1993). As depicted in Figure 4C, there are four response types organized into three sagittal zones. In the most medial zone, CSA responds most stronglyto optic flow resulting from translation backwards along an horizontal axis 45° to the midline such that there is a focus of contraction at 45° contralateral azimuth (contraction neurons). Medial to this is a zone where the CSA responds most strongly to optic flow resulting from either (i) forward translation along an horizontal axis 45° to the midline such that there is a focus of expansion at 45° ipsilateral azimuth, or (ii) upward translation along the vertical axis. Finally, lateral to this is a zone where the CSA responds to the optic flow resulting from downward translation along the vertical axis (Wylie et al., 1998; Graham and Wylie, 2012). Thus, the VbC is well suited to analyze the optic flow resulting from self-translation and self-rotation as birds fly through the world. This is combined with vestibular information derived from separate end organs for analyzing rotation (semicircular canals) and translation (otolith organs) (Pakan et al., 2008).

## The optic flow pathway to the oculomotor cerebellum

A second optic flow pathway to the cerebellum of birds is to folia VI-VIII of the posterior lobe, which are collectively known as the “oculomotor cerebellum” (OCb; Voogd and Barmack, 2006). This region of the cerebellum has been implicated in flight insofar as the “strong fliers” as defined by Larsell (1967) tend to have a large posterior lobe, in particular folia VI and VII, and these folia are significantly smaller in flightless birds (Iwaniuk et al., 2007). The LM and nBOR project as MFs to folia VI-VIII (Clarke, 1977; Brecha et al., 1980; Figure 4D). The projection from nBOR is relatively small, but the projection from LM is massive and originates mainly in LMm (~75%) (Pakan and Wylie, 2006). As shown in Figure 4D, folia VI-VII are part of a much more extensive visuo-motor network that incorporates visual information from several indirect sources. These folia also receive heavy MF projections from the medial and lateral pontine nuclei (MP, LP) (Brodal et al., 1950; Freedman et al., 1975; Clarke, 1977; Pakan and Wylie, 2006). The pontine nuclei in turn receive projections from several retino-recipient sources including nBOR (although this is minor; Wylie et al., 1997), the optic tectum, which sends projection mainly to LP (Hunt and Künzle, 1976; Wylie et al., 2009), and LM, which projects mostly to MP (Clarke, 1977; Gamlin and Cohen, 1988a), and Glv (Marín et al., 2001). Glv itself receives input from the optic tectum (Hunt and Künzle, 1976; Wylie et al., 2009; Vega-Zuniga et al., 2014), and in turn projects heavily to LM, mainly to LMm (Vega-Zuniga et al., 2016), providing some interconnectivity among these retinorecipient regions before sending efferents to MP and folia VI-VIII. Because this system receives direct and indirect input from several visual regions, it is well suited for integrating different types of visual information. Whereas the LM is concerned with optic flow, neurons in the tectum respond to small moving stimuli (Frost and Nakayama, 1983; Frost et al., 1990). The exact nature of visual processing in Glv remains elusive, however, electrical stimulation of the Glv elicits discrete, precise orienting head movements (Vega-Zuniga et al., 2011). All of this information is integrated in the pontine nuclei and folia VI-VIII, but for what purpose?

The integration of local motion and optic flow information in primate visual cortex is implicated in “steering” to avoid obstacles during locomotion through cluttered environments (Page and Duffy, 2008; Elder et al., 2009). Similarly, Hellmann et al. (2004) suggested that the tecto-pontine pathway in birds is involved in avoidance behavior. Thus, perhaps this network integrating optic flow and local motion signals is important for obstacle avoidance as birds fly through cluttered environments. For example, during translation, a radial optic flow pattern would result, which would be detected by neurons in LM and nBOR. In addition, self-motion would cause motion parallax of stationary objects at different depths. Such local motion relative to the background, is the ideal stimulus to activate deep tectal cells (Frost and Nakayama, 1983), which project to the pontine nuclei (Hellmann et al., 2004). This combination of local motion and optic flow during self-translation would then be analyzed by the pretecto-ponto-cerebellar system to folia VI-VIII (Figure 4D), allowing a flying bird to then adjust its direction and velocity accordingly.

## Optic flow pathways to the cerebellum of mammals

The visual pathways that convey optic flow information to the cerebellum of mammals are similar to those in birds and have been studied extensively (Simpson, 1984; Voogd and Barmack, 2006). A detailed review of this literature is beyond the scope of this review but a brief summary follows. As in mammals (and other vertebrates), optic flow is analyzed in specialized optic pathways, which begin in two retinorecipient nuclei, the medial and dorsal terminal nuclei of the AOS (homolog to nBOR of birds) and the nucleus of the optic tract (NOT; homolog to LM of birds). These two nuclei are also highly conserved among other vertebrates (Simpson, 1984; McKenna and Wallman, 1985a). Similar to birds, in mammals two regions of the cerebellum ultimately receive visual inputs from these two nuclei, the oculomotor cerebellum (folia VI-VIII) and the VbC (Reviewed in Voogd and Barmack, 2006). However, in contrast to birds, in most mammals optic flow information does not reach the cerebellum directly as mossy fibers from the terminal nuclei and NOT, but rather indirectly through different relay nuclei (see Simpson, 1984; Pakan et al., 2010). As in birds, these nuclei do project to regions of the inferior olive which then projects as climbing fibers to the VbC (Giolli et al., 2006). In the case of the oculomotor cerebellum of mammals, visual projections arise from the pontine nuclei, which like in birds receive projections from the accessory optic system, the NOT, the ventral geniculate, pretectum and the superior colliculus (reviewed in Voogd and Barmack, 2006). The existence of very similar optic flow pathway to the cerebellum of mammals strongly suggest that these are ancestral characters of at least all land vertebrates, and that like the expansion of the cerebellum, they precede the evolution of flight in birds.

## Concerted evolution of the LM with other visual nuclei

**Figure 5:**
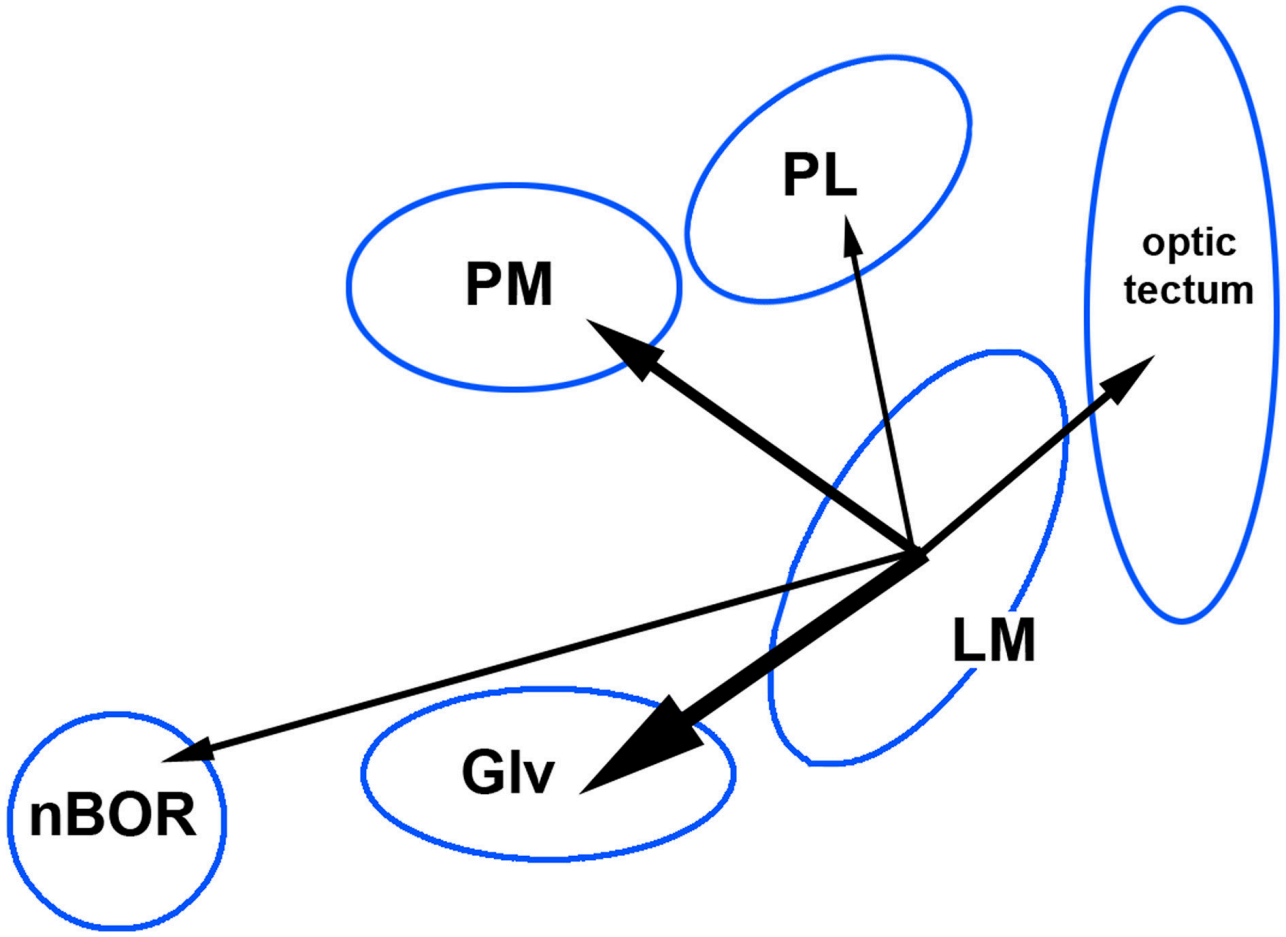
Correlations of the relative size of the pretectal nucleus mesencephali (LM) with the medial and lateral pontine nuclei (PM, PL) and other visual nuclei including the nucleus of the basal optic root (nBOR), the ventral lateral geniculate nucleus (Glv) and the optic tectum. The size of the arrows represents the strength of the correlation. These data are from Gutiérrez-Ibáñez et al. (2014); Gutierrez-Ibanez et al. (2017). See text for details.

Jerison's principle of proper mass states that an increase in the size of a brain nucleus is associated with an increase in the processing power needed to meet behavioral requirement (Jerison, 1973). The classic example is the increased size of the hippocampus in food caching birds (Sherry et al., 1989). Iwaniuk and Wylie (2007) suggested the increase in size of the LM in hummingbirds and other birds is related to an increased need for stabilization during hovering. Although through evolution a single brain nucleus can increase (or decrease) in size, so called “mosaic evolution,” more often large parts of the brain or whole systems evolved together: “concerted evolution” (Striedter, 2005). Gutiérrez-Ibáñez et al. (2014) showed in birds that an increase in size of one visual nucleus is associated with increased in other visual nuclei, but the strength of the correlations varied greatly across pairs of visual nuclei. This is shown in Figure 5, where we show the correlation of the relative sizes of various visual nuclei across 77 species of birds (data are from Gutiérrez-Ibáñez et al. (2014), plus unpublished measurements of pontine nuclei). Note that while the correlation between the LM and nBOR is of a significant magnitude (0.25), the correlation is higher between the LM and PM (0.39) and highest between LM and Glv (0.62). The correlations between LM and PL and LM and the optic tectum are also comparatively low (0.2 and 0.29, respectively). The correlation between PM and Glv is also high (0.54). Thus, the LM tends to increase in size with Glv and the PM, more so than other structure shown in Figures 4A,D. This covariation likely reflects the aforementioned connections among these nuclei and that these regions evolve in a correlated fashion due to increased information processing requirements of one or all nuclei. Based on our findings in hummingbirds, we propose that LM may be the region undergoing direct selection and that the associated changes in Glv and PM are through correlated evolution, as a result of increasing input and output requirements of LM. This preliminary hypothesis could be tested using evolutionary path analysis (von Hardenberg and Gonzalez-Voyer, 2013) to tease apart directionality of correlated size changes.

## Hypertrophy of the LM for the analysis of optic flow and motion parallax

We have established that LM is enlarged in hummingbirds and, as depicted in Figure 4, that the LM feeds two cerebellar pathways. One is to the VbC (IXcd) that arises mainly from LMl and also from nBOR (Figure 4A). The second is to folia VI-VIII where there is an integration of optic flow signals from LMm and local motion from a tecto-pontine circuit and involving Glv (Figure 4D). We suggest that the hypertrophy of LM in hummingbirds may be due to an expansion LMm given the following facts.

- Across birds, an increase in the size of the LM is associated with increases in the size of Glv and the PM, more so than nBOR (Figure 5).
- Related, the Glv projects mainly to LMm as opposed to LMl (Vega-Zuniga et al., 2016).
- Some neurons in LM prefer fast speeds, whereas others prefer slow speeds. Invariably, the slow neurons prefer temporal-to-nasal motion, whereas the fast neurons prefer various direction of motion (Wylie and Crowder, 2000). As most neurons in LMl prefer temporal-to-nasal motion, one can infer that the fast and slow neurons are more associated with LMm and LMl, respectively.
- Xiao and Frost (2013) showed that the fast LM neurons respond best to motion parallax.
- Finally, Gaede et al. (2017) showed that neurons in LM of hummingbirds respond differently than other species in two respects. First, there was no bias to neurons that prefer temporal-to-nasal motion, as has been observed in the LM of other birds. Second, the LM neurons in hummingbirds preferred faster velocities than other birds. Indeed, the LM neurons in hummingbirds were rather tightly tuned to fast velocities. This bias away from neurons that prefer temporal-to-nasal motion, and toward fast velocities suggests that the hummingbird LM is processing stimuli typically processed by the LMm in pigeons.

As previously mentioned, Iwaniuk and Wylie (2007) suggested that the hypertrophy of LM in hummingbirds is driven by the increased need to use optic flow to drive the optokinetic response, which facilitates stabilization during hovering. This argument was supported by an expansion of LM in other birds capable of hovering (e.g., kingfishers), although the increase in the size of LM in these species was smaller than in hummingbirds. However, Ibbotson (2017) suggested that the selectivity of hummingbird LM neurons to fast velocities may be related to flying in dense vegetation, as images that are close to the animal will move very quickly across the retina. The five points laid out above suggest that the expansion of LM in hummingbirds could be driven by a differential increase in the size of LMm, where neurons tend to be tuned to higher speeds (Winterson and Brauth, 1985) but also motion parallax, which would occur as they fly through cluttered environments. As Iwaniuk and Wylie (2007) measured LM as whole, there is no evidence that the expansion of LM in hummingbirds is due to a relative increase in the size of LMm vs. LMl. It would be interesting to see if this is the case and if hummingbirds show an increase in the magnitude of the projection from the LMm to the oculomotor cerebellum. In summary, the hypertrophy of LM in hummingbirds may not be related to hovering alone, but could also be related to an increase in the processing demands associated with the pathway to the oculomotor cerebellum as they fly through a cluttered environment while feeding.

## Conclusions

In this paper we have reviewed evidence that visual cerebellar pathways are involved in the control of flight. Moreover, there are two pathways involved: a pathway to the VbC involved in the processing of optic flow resulting from self-translation and self-rotation, and a pathway to folia VI-VIII that integrates optic flow and local motion information such as that which occurs during self-motion through cluttered environments. These pathways are fed by different parts of the LM, LMl, and LMm, that have different response properties to optic flow stimuli. Further, we suggest that the hypertrophy of the LM observed in hummingbirds may be driven more so by an hypertrophy of the LMm to support an increased demand of integrating local motion and optic flow signals to process motion parallax in cluttered environment. Hummingbirds spend much of their time feeding, and clearly they will be faced with optic flow and motion parallax as they forage through a patch of flowers. In this respect, we completely concur with Ibbotson (2017) who stated: “It is likely that their [hummingbirds] habit of flying close to flowers in dense vegetation has tuned the hummingbird LM to its specific visual environment.” Hummingbirds may, in fact, be an especially powerful group in which to gain a deeper understanding of the role that optic flow processing plays in flight behavior of birds.

## Author contributions

All authors had full access to all of the data in the manuscript and take responsibility for the integrity of the data and the accuracy of the data analysis. Conceived and designed the manuscript: DW. Performed experiments and acquired data: CG-I, AI, and AG. Data analysis: CG-I, AI, and AG. Writing the manuscript: DW, CG-I, AI, and DA. Supervised the study: DW, DA, and AI.

### Conflict of interest statement

The authors declare that the research was conducted in the absence of any commercial or financial relationships that could be construed as a potential conflict of interest.
